# Pride Camp: Pilot study of an intervention to develop resilience and self-esteem among LGBTQ youth

**DOI:** 10.1186/s12939-021-01488-1

**Published:** 2021-06-29

**Authors:** Lance S. Weinhardt, Linda M. Wesp, Hui Xie, Jennifer (Jen) Murray, Jeanette Martín, Sarah DeGeorge, Caleb B. Weinhardt, Maren Hawkins, Patricia Stevens

**Affiliations:** 1 Joseph J. Zilber School of Public Health, University of Wisconsin-Milwaukee, Milwaukee, WI USA; 2 College of Nursing, University of Wisconsin-Milwaukee, Milwaukee, WI USA; 3 Lesbian, Gay, Bisexual Transgender (LGBT) Resource Center, Division of Student Affairs, University of Wisconsin-Milwaukee, Milwaukee, WI USA; 4 Journalism, Advertising, and Media Studies, University of Wisconsin-Milwaukee, Milwaukee, WI USA; 5 Lewis & Clark College, Portland, OR USA

**Keywords:** Youth camp, Resilience, Community connections, LGBTQ youth, Transgender youth

## Abstract

**Background:**

Many LGBTQ youth experience rejection and discrimination in their families and schools, and the range of interventions for improving their resilience and well-being is limited. We developed and piloted an LGBTQ-youth-focused intervention to build resilience and promote health equity, called Pride Camp, in an urban environment in the Midwest.

**Methods:**

Using a mixed-method approach we examined the impact of Pride Camp on resilience and other measures of well-being among LGBTQ high school students who attended camp on a college campus in 2015, 2016, and 2017. Camp attendees and the research sample included a majority proportion of transgender and gender nonbinary (TGN) youth.

**Results:**

Pre- and post-test data from our quantitative surveys (*n* = 28), indicated significant increases in resilience, self-esteem, and quality of life in LGBTQ youth who attended camp. Similar results were found among the TGN participants (*n* = 19). Qualitative data from focus groups indicated that specifically for TGN youth, the affirming environment at the camp provided social opportunities that they had not found elsewhere.

**Conclusions:**

Findings suggest that the Pride Camp intervention provides a platform for LGBTQ youth to meet peers and engage in LGBTQ communities, improving their resilience and outlook on the future. A larger controlled study of the Pride Camp intervention including measurement of additional specific health outcomes over a longer follow-up period is warranted to examine the impact of this program on health equity.

## Introduction

It is imperative to understand how to shape our schools, communities, and society to be more inclusive and empowering, as more people are openly identifying and expressing their sexual orientation and gender diversity at young ages. We define inclusive here as an environment that welcomes all sexual orientations and gender identities. A nationally representative study of LGBTQ secondary school students conducted in 2011 used structural equation modeling (SEM) to show significant associations between unwelcoming campus environments and educational attainment and mental health [1]. Characteristics of unwelcoming campus environments, included a lack of inclusive school policies, supportive school personnel, and Gay-Straight Alliance organizations; and the presence of active discrimination, micro-aggressions, harassment, and violence [1]. Undesirable environments for LGBTQ youth increase detention rates, mental health difficulties, and reduce social connections with others [2, 3].

Among transgender and non-binary students (TGN), the National School Climate Survey conducted in 2015 found that TGN students faced more hostile school climates, including non-inclusive policies, lack of inclusive curricular resources, and greater victimization, when compared to their cisgender LGBQ peers [1]. In another large study, TGN youth were four times more likely to experience bullying in school, and bullying (mental and physical harassment and aggression) was associated with 3-fold greater odds for substance use, depression, and anxiety among transgender youth compared to non-transgender youth [4]. This has long-term avoidable and unjust consequences for TGN individuals. Roughly 50% of transgender adults in the U.S. reported experiencing negative interactions and lack of support from caregivers during childhood or adolescence [5]. TGN adults who faced rejection from their families were twice as likely to have experienced homelessness (40%), twice as likely to have engaged in sex work (16%), and more likely to have attempted suicide (49%) compared to non TGN individuals [5].

LGBTQ youth experience unique difficulties in schools and among their families. Thus, immersive interventions such as overnight summer camps tailored for LGBTQ youth may be an effective setting for building life skills and resilience through the creation of an inclusive and affirming environment [6]. The few previous camps designed for LGBTQ youth reported in the literature indicate that this is a promising approach [7, 8]. Perhaps the most well-established camp is Camp fYrefly (“fostering Youth, resilience, energy, fun, leadership, yeah”) in Canada [7]. Camp fYrefly is offered by the Institute for Sexual Minority Studies and Services at the University of Alberta. The camp was founded in 2004 and has expanded to several different locations across Canada. Fyrefly is a traditional outdoor summer camp experience, focused on LGBTQ youth leadership, based on research and pedagogy that creates an inclusive climate. Camp fYrefly is structured around five themes: Creating a socially just and inclusive community; growing into resilience and youth leadership capacity; helping youth to know their rights as a basis for empowering them to address bullying, learning through art, music, writing, visual and performing arts, and games; and self and social development. Grace and Wells describe campers’ reactions to the camp environment, noting that it is a profoundly positive experience for them [7].

Gillig, Miller, and Cox describe the impact of another intervention called Brave Trails, an outdoor summer camp model, adapted specifically for building strengths and reducing depressive symptoms in LGBTQ youth ages 12–20, and the first camp for LGBTQ youth in the United States [8]. In an uncontrolled pilot test, they found that campers (*n* = 56) reported increased hope, identity affirmation, and a reduction in depressive symptoms, from pre to post test. They also administered a parent survey and found that parents perceived similar positive changes in their children after attending camp. This study focused on individual changes in the campers, and parent-perceptions of the changes, rather than focusing on the environmental conditions of the camp. Hence, the present study, which also emphasized environmental conditions, provides a unique contribution, in that we explore how environmental conditions impact individual well-being.

In contrast to these previous camps designed for LGBTQ youth, which have primarily been set in nature, Pride Camp was situated on a college campus in an urban setting. Our program was designed to build resilience, self-esteem, leadership skills, and other personal strengths, while also encouraging LGBTQ high school students to envision themselves as future college students. Programs such as ours and the aforementioned camps, when implemented widely, could potentially improve the long-term health of LGBTQ-youth and reduce health disparities, thereby promoting health equity, in this community.

### Theoretical framework

Meyer’s minority stress model provided the theoretical framework for the study. Minority stress suggests that stigma, prejudice, and discrimination negatively contribute to the overall health and well-being of an individual [9, 10]. Prejudice and discrimination are connected to increased vigilance for future stigmatization, concealment, and internalized stigma. Meyer suggests two levels of intervention derived from the theory: individual-level intervention, to improve a person’s means of evaluating their situation and improve coping, and societal level changes, which reduce exposure to stress by changing the larger social environment [10].

Hendricks and Testa further developed Meyer’s theory for transgender and gender non-conforming people with the Gender Minority Stress and Resilience (GMSR) model [11]. GMSR theory illustrates that gender minority stressors, such as gender-related discrimination, victimization, and rejection, negatively impact health and well-being; while resiliency factors, such as community connectedness, social support, and pride, can serve as buffers against stressors [11, 12]. According to the American Psychological Association (APA), broadly defined, self-esteem is defined as “the degree to which the qualities and characteristics contained in one’s self-concept are perceived to be positive.” [13] While resilience is defined as “the process of adapting well in the face of adversity, trauma, tragedy, threats, or significant sources of stress.” [14] Hendricks and Testa do not explicitly define resilience, rather they draw upon the literature to discuss factors that enhance resilience, summarized above as community connectedness, social support, and pride. Hendricks and Testa draw upon Singh and McKleroy, who note that “pride in one’s gender and ethnic/racial identity … recognizing and negotiating gender and racial/ethnic oppression … navigating relationships with family … accessing health care and financial resources … connecting with an activist transgender community of color and cultivating spirituality and hope for the future” are factors that can help build resilience. [11, 15] In essence, research based on the GMSR model among transgender youth indicates that transgender youth face disproportionate rates of bullying and harassment compared to non-transgender peers. These experiences are significantly associated with substance use behaviors [4]. Research specifically examining resilience and the role of social support in the lives of transgender youth is currently lacking, with few exceptions [16, 17], and very few interventions have been tested [7, 8].

The Minority Stress Model [10], and GMSR [11], informed the development of Pride Camp as an intervention for LGBTQ youth. The goal was to create an affirming environment that offered experiences of community connectedness, social support, and pride, as well as opportunities to learn coping, resilience skills, and foster self-esteem.

### The current study

This research served as an uncontrolled pilot test of the effects of a six-day on-campus program, called Pride Camp, on resilience and well-being among LGBTQ youth. This pilot research examined LGBTQ youth using two approaches: 1) we aggregated Pride Camp data from 2015 to 2017 to assess whether there were significant changes in psychological outcomes between pre- and post-intervention, and, because our sample consisted of a large proportion of TGN youth, in sub-analysis we examined whether these changes were present for the TGN campers specifically, and 2) we conducted two semi-structured focus groups in 2016 to describe the experiences of TGN campers at Pride Camp. The two datasets provided complementary mixed-method approaches to understanding the possible impact of Pride Camp in promoting identity development, resilience, and social engagement for LGBTQ youth, with additional focus on the experiences of TGN campers.

### Positionality

Due to the content of this study, it is necessary to explicate the positionality of the authors of this manuscript. Some, but not all, of the authors are LGBTQ identified. Authors included current faculty at university, as well as undergraduate and graduate students. Three of the authors worked at the Lesbian, Gay, Bisexual Transgender (LGBT) Resource Center, Division of Student Affairs at their university, at the time of this study. The co-authors include people who conceived the study and helped to collect the data, some who worked on initiating and developing Pride Camp prior to it being evaluated (it was conducted as a summer event for youth for 1 year prior to any evaluation occurring), and some who were involved in the conduct of the camp during the years it was conducted but not involved in data collection.

### Consent

For both the qualitative and quantitative aspects of the study, as detailed below, prior to beginning the study, informed written consent was obtained from the campers and their legal guardians.

## Methods

### Study 1: Quantitative study

#### Participants and procedure

The Pride Camp intervention was advertised through Pride events, GSAs (gay–straight alliance / gender and sexuality alliance) at area high schools, social media, and through personal and professional contacts in an urban environment in the Midwest. The eligibility criteria to participate in the research element included: 1) Age between 13 and 17 years; 2) Attendance at Pride Camp; 3) Self-identification as LGBTQ; and 4) Informed consent from both the camper and a legal guardian. People attending Pride Camp were not required to participate in the research component. The camp took place on a urban public research university campus in the Midwest. The study was approved by the appropriate Institutional Review Board.

Of a total sample of 42 camp attendees between 2015 and 2017, 9 were returners (we use only pre-post data from a camper’s first year of attendance), and 5 campers did not provide complete written informed consent; therefore, data from 28 campers were analyzed for the study. As shown in Table 1 with other demographic information, among these 28 campers, 19 were non-Hispanic Whites, 1 was Hispanic, 1 was Black, 1 was Asian American, 1 was Native American, and 6 self-identified as multiracial. Twenty-three campers were assigned female sex at birth (5 cisgender women, 8 transgender men, 5 non-binary-identified, and 4 multiple identities) and 5 were assigned male sex at birth (4 cisgender men and 1 transgender woman). Thus, 68 % (*n* = 19) of campers were non-cisgender-identified. The average age of the campers was 15.8 years (*SD* = 1.06).

**Table 1:** Descriptive statistics, Pride Camp 2015–2017 (*N* = 28)

Variable	N (%) / Mean (SD; range)
Age	15.8 (1.06; 14–18)
Race/ethnicity	
White	19 (67.9)
Hispanic or Latino	1 (3.6)
Black or African American	1 (3.6)
Native American or American Indian	1 (3.6)
Asian/Pacific Islander	1 (3.6)
Multiracial	6 (17.9)
Residency	
Urban	11 (39.3)
Suburban	14 (50.0)
Rural area	1 (3.6)
Other	2 (7.1)
Assigned sex at birth	
Male	5 (17.9)
Female	23 (82.1)
Gender Identity	
Man/boy	5 (17.9)
Woman/girl	5 (17.9)
Transgender	8 (28.6)
Non-binary/GNC	6 (21.4)
Multi-identity	4 (14.3)
Sexual Orientation	
Lesbian/gay/homosexual	6 (21.4)
Straight/heterosexual	1 (3.6)
Bisexual/pansexual/Polysexual	14 (50)
Questioning	1 (3.6)
Queer	1 (3.6)
Asexual	1 (3.6)
Multi-identity	4 (14.3)

#### Pride camp intervention

The intervention we studied (referred to as Pride Camp) was initiated and designed to inspire LGBTQ high school students to build resilience and improve perceived quality of life by embracing their identities and gaining a better understanding of marginalized identities through community building, self-expression, and connections in the community. The Camp was designed and implemented by LGBTQ staff of the campus LGBTQ resource center. During the 6 days of Pride Camp, campers participated in sessions addressing a range of topics, including social justice (e.g., structural, interpersonal, and internal racism, heterosexism, cisgenderism, and ableism), medical health resources (e.g., preventive health, gender affirming hormone therapy), careers (e.g., academic options and life path), journaling and developing a personal narrative (e.g, exploring and/or affirming identity development), LGBTQ history, healthy lifestyles (e.g., self-help, coping strategies), and spirituality. Each session was highly structured and facilitated by people from LGBTQ-related communities or professional services. Additionally, each session was designed to be educational, entertaining, and culturally sensitive. Sessions involved group discussions, videos, games, brainstorming, experiential exercises, and skill-building activities. Each session focused on incorporating the theme of “embracing multiple and intersecting identities,” which encouraged the campers to understand and embrace their identities, behave responsibly for their own benefit, and foster self-growth for the future.

During the intervention, peer counselors stayed in rooms adjacent to campers in a student residential hall on campus. Peer counselors were college students or staff from the LGBTQ student resource center on the campus. They were selected based on their LGBTQ youth-related work experiences via interviews. There were 4–5 peer counselors each year of the camp. Each underwent a week of intensive training prior to the camp, by the LGBT-resource-center developers of the camp. Each peer-counselor was LGBTQ identified. Additionally, peer counselors participated in a 2-day intensive leadership training course on the basic skills of small-group facilitation. Their main responsibilities included: 1) Supporting facilitators to involve campers in sessions; 2) Providing peer support in the form of nonjudgmental assistance to campers when articulating personal goals and self-growth through individual and group sessions; 3) Assisting campers in building social skills in the community; and 4) Providing psychological support when needed.

Pride Camp provided LGBTQ youth education and community building at different levels. For instance, on the level of policy and structure, Pride Camp provides gender neutral bathroom facilities [6]. On the interpersonal level, all campers and peer counselors are LGBTQ-identified. In other intimate settings, campers may have concerns about privacy or fear other campers’ perceptions of their gender or sexual identities. Therefore, living and interacting with those who share similar identities offers the opportunity to build confidence and a sense of belonging. Moreover, some TGN youth do not feel the freedom to travel to and participate in recreational programs or sports due to fear of verbal and physical harm, and traditional gender-based recreational programming may not be interesting to them nor meet their needs for self-expression [18]. Because of the unique environment of the camp, their experiences and identity are likely to be validated and supported.

#### Measures

Quantitative data collection occurred at the beginning of camp, before any structured sessions, and post-test data were collected after the final programmed session of camp. Data were collected via confidential self-administered online surveys, assessing campers’ demographic characteristics, self-esteem, perceived social support, perceived discrimination, resilience, and quality of life.

Campers were asked about their race/ethnicity, age, living environment/situation, assigned sex at birth, gender identity, sexual orientation, and GSA involvement. Two questions about gender identity were asked, both with multiple and open-ended options where campers could provide the best fitting response. The first question was “What is your current gender identity?”. The responses included: 1) Man/boy; 2) Woman/girl; 3) Genderqueer, neither exclusively male nor female; and 4) Additional gender category, please specify. Further, to articulate individual gender identity and whether it corresponds to their assigned sex at birth, another self-identification question was prompted to exclusively capture their transition status or non-cisgender identity (e.g., agender, transgender man, transgender woman, gender nonconforming, genderqueer, non-binary, other).

*Self-Esteem* was assessed using the 10-item Rosenberg Self-Esteem Scale [19]. Participants responded to questions on a 4-point Likert scale ranging from 1 (*strongly agree*) to 4 (*strongly disagree*). An example item was “On the whole, I am satisfied with myself”. The sum scores were yielded across 10 items, where the high scores reflected high self-esteem. The validity of the instrument was evaluated and found to be acceptable in adolescents [19], and stress-related research [20, 21]. In the present study, RSES had a high internal consistency (Cronbach’s α = 0.88).

*Social Support* was assessed by a 12-item Multidimensional Scale of Social Support (MSPSS) [22]. The MSPSS has been used in studies of LGBT youth [16], with Chronbach’s alpha = .89. The MSPSS has three 4-item subscales: Significant Other (SO), Family (F), and Friends (FR). Sample items for each subscale include: “My family tries to help me,” “I can talk about problems with my friends,” and “There is a special person who is around when I am in need.” All items were reverse-scored so that higher scores indicated more perceived support. For the present study, the MSPSS has strong internal consistency (α = 0.90) for the overall scale and subscales (0.87–0.94).

*Resilience* was measured by a 25-item Resilience scale [23], using a 7-point Likert scale from 1 (*strongly disagree*) to 7 (*strongly agree*). An example item was “When I make plans I follow through with them.” The RS is well-adapted to evaluate resilience in the adolescent population due to good psychometric properties [24–26]. The sum scores range from 25 to 175 with higher scores reflecting higher resilience. The internal consistency was high in the present study (Cronbach’s α = 0.91).

*LGBT Stigma* was measured by a revised sexual stigma scale from Logie and Earnshaw [27], which was designed to assess frequencies of experienced discrimination among TGN youth, including stereotypes, enacted stigma, and harassment. We added two items related to stigma or discriminatory experiences in schools and public facilities, and removed two items that were not relevant to youth. This is a 12-item, 4-point Likert-type scale ranging from 1 (Many times) to 4 (Never). After conducting an exploratory factor analysis using principal components analysis with varimax rotation, 12 items were exclusively loaded on two factors, consistent with the analysis of the original scale: perceived stigma and enacted stigma [27]. *Perceived Stigma* (6 items) reflected experiences of perceived or normative stigma (e.g., hearing or feeling social devaluation of queer, lesbian, and bisexual women), which included such statements as “How often have you heard that LGBT+ people are ‘not normal?’” Another factor is *Enacted Stigma* (6 items), which referred to the tangible behaviors and interactions of discrimination, hate, prejudice, or stigma from others; one such item is “How often have you been harassed by teachers, school staff, or police for being LGBT+?” All items were reverse scored so that higher scores indicated greater perceived stigma. The internal reliability for Perceived Stigma Subscale was 0.87 and 0.74 for Enacted Stigma Subscale.

*Quality of Life* was assessed by the Youth Quality of Life-Short Form (YQoL-SF 2.0) [28, 29], which included four domains: 1) sense of self; 2) social relationships; 3) culture and community environment; and 4) general QoL [30]. Response options ranged from 0 (*Not at all*) to 10 (*Very much*). An example item is “I am able to do most things as well as I want.” Fifteen transformed item scores were calculated and the mean of these items formed the total score. Higher scores indicated higher quality of life. The YQoL-SF 2.0 had good internal consistency in this study (Cronbach’s α = 0.84).

#### Statistical analysis

Before conducting statistical analyses, we examined missing data patterns and mean-imputed variables with 2% of values missing at random or less. Descriptive analyses were conducted, followed by paired t-tests to assess the differences in self-reported psychological outcomes between baseline and post-intervention. Analyses were conducted using STATA version 14.

### Quantitative results

There were three significant differences in psychological outcomes between baseline and post-intervention for the total sample, as shown in Table 2. Self-esteem significantly increased from pre- to post-intervention (means, 22.68 vs. 25.04; *p* = 0.0002; Cohen’s *d* = 0.77). Resilience (means, 118.56 vs. 125.61; *p* < 0.003; Cohen’s *d* = 0.53) and YQoL (means, 53.29 vs. 59.05; *p* < 0.001; Cohen’s *d* = 0.55) also significantly increased after the intervention compared to the baseline. There were no significant changes observed in social support, perceived stigma, or enacted stigma (LGBTQ stigma) between baseline and post-intervention.

**Table 2:** Changes in outcome variables, Pride Camp 2015–2017 (*N* = 28)

Measures	Pretest Range	Pretest Mean (SD)	Posttest Range	Posttest Mean (SD)	t	*p*	Effect Size (d)
RSES	11–37	22.68 (5.08)	18–38	25.04 (3.83)	−4.06	0.0002	0.77
Significant other ^a^	12–28	22.07 (4.82)	12–28	22.15 (4.84)	−0.08	0.94	0.02
Family ^a^	7–26	18.11 (5.58)	8–28	18.46 (5.30)	−0.55	0.59	0.06
Friend ^a^	11–28	21.79 (5.39)	14–28	22.14 (4.21)	−0.40	0.70	0.07
Perceived stigma ^b^	1.17–3.67	2.16 (0.76)	1.17–4.00	2.18 (0.80)	−0.18	0.856	0.03
Enacted stigma ^b^	2.17–4.00	3.01 (0.58)	1.83–4.00	3.01 (0.61)	0.00	1.00	0.00
Resilience	57–164	118.56 (20.15)	70–159	125.26 (18.04)	−3.27	0.003	0.53
YQoL ^c^	27.33–79.33	53.28 (13.36)	39.33–89.33	59.05 (12.82)	−3.73	.001	0.55

The observed score here is at the lower end of the range of scores reported in Patrick, consistent with LGB students who had been bullied because of perceived sexual orientation. Scores are comparable between studies because the total scale score on the YQoL is the total of transformed item scores divided by the number of items

^a^ Subscales of MSPSS

^b^ Subscales of LGBT Stigma

^c^ Patrick et al. used a 6-item version of this scale in a large sample of high-school age LGB youth (2002). They reported scores across different categories of participants (by grade, by gender, and by whether or not they were bullied due to perceived sexual orientation or other factors). QoL scores ranged from 54 to 83 across these different combinations of categories

Among the 19 TGN campers, results were similar. As shown in Table 3, Self-esteem increased from pre to post intervention (means, 22.15 vs. 24.53; *p* = 0.004; Cohen’s *d* = 0.69), as did YQoL (means, 51.85 vs. 56.94; *p* < 0.005; Cohen’s *d* = 0.66). Meanwhile, Resilience (means, 120.44 vs. 125.11; *p* < 0.062; Cohen’s *d* = 0.43) increased with a medium effect size, but the significance level was not below .05. As with the overall sample, no significant change observed in social support, perceived stigma, or enacted stigma (LGBTQ stigma) between baseline and post-intervention.

**Table 3:** Changes in Outcome Variables for Transgender and Nonbinary Youth Only (*n* = 19)

Measures	Pretest Range	Pretest Mean (SD)	Posttest Range	Posttest Mean (SD)	t	*p*	Effect Size (d)
RSES	11–37	22.15 (5.39)	18–38	24.53 (4.25)	−2.98	0.004	0.69
Significant other ^a^	12–28	22.15 (4.88)	13–28	22.00 (4.88)	0.17	0.56	0.04
Family ^a^	8–26	18.89 (4.78)	8–28	19.47 (4.96)	−1.29	0.11	0.29
Friend ^a^	12–28	21.26 (5.06)	14–28	22.36 (4.52)	−0.12	0.45	0.04
Resilience	57–164	120.44 (21.68)	70–159	125.11 (19.33)	−1.61	0.062	0.43
YQoL ^c^	27.33–74.00	51.85 (13.04)	41.33–89.33	56.94 (12.89)	−2.88	.005	0.66

The observed score here is at the lower end of the range of scores reported in Patrick, consistent with LGB students who had been bullied because of perceived sexual orientation. Scores are comparable between studies because the total scale score on the YQoL is the total of transformed item scores divided by the number of items

^a^ Subscales of MSPSS

^b^ Subscales of LGBT Stigma

^c^ Patrick et al. used a 6-item version of this scale in a large sample of high-school age LGB youth (2002). They reported scores across different categories of participants (by grade, by gender, and by whether or not they were bullied due to perceived sexual orientation or other factors). QoL scores ranged from 54 to 83 across these different combinations of categories

### Study 2: Qualitative focus groups

To further understand the experiences of TGN campers and the potential benefits of Pride Camp for TGN youth, and because we had a predominance of TGN participants in this project, we conducted two focus groups in 2016. The focus groups enabled us to ask questions about experiences at camp and explore the impact for TGN youth specifically.

#### Participants & procedure

Eight transgender or gender non-binary campers who participated in study 1 (quantitative phase) in 2016 (gender identities were self-selected via the survey at baseline) were randomly assigned to two focus groups conducted toward the end of Pride Camp. Before data collection, written informed consent was obtained from the teens and their legal guardians (the same consent form used in Study 1). Seven of these campers were non-Hispanic White; one was biracial. All campers were assigned female sex at birth, with current gender identities self-described as transgender, genderqueer, or non-binary.

The semi-structured focus groups were facilitated by an experienced qualitative researcher and attended by a student member of the research team. Each focus group was audio recorded and lasted about 2 h. We began the group discussion by introducing the general topic of participants’ feelings and experiences from the last couple of days of camp. We followed this with questions about what campers liked and did not like about camp.

#### Qualitative data analysis

Audio recordings from the focus groups were transcribed and qualitative analysis was conducted using thematic analysis [31]. Thematic analysis was chosen to identify major themes about TGN youth experiences at Pride Camp so that we could better understand the ways that Pride Camp impacted TGN youth. First, pre-coding was conducted to identify initial impressions about main concepts youth used to describe their experiences at camp, and challenging or helpful components of camp. An initial set of codes was developed and applied to the entire transcript, followed by categorization and organization of these codes into final themes and subthemes that encapsulated the meaning of the initial codes [31]. Exemplar excerpts are provided below to further illustrate the meaning of the themes.

Overall, our analysis identified two main themes that explain how TGN youth experienced Pride Camp. Theme 1 was “Group Energy is Something Special” and Theme 2 was “An Affirming Environment Created a Safe Space.” Several sub-themes under Theme 2 described specific examples of ways youth were able to learn and grow in an affirming and safe space. These examples are discussed in more detail below.

#### Qualitative results

##### Theme 1: group energy is something special

TGN campers in both focus groups identified a common experience of feeling “something special” while at camp. They described feeling an “energy” that was unfamiliar and “refreshing.” TGN youth described this energy as “something special” because they were often unable to be themselves outside of camp, especially when in peer groups. While at camp they were met with acceptance, which allowed them to be exactly who they are without hiding anything. One participant said:

“I think I found that there aren't very many places where I feel like I can be all of myself, because I feel like there are parts of my past or my identity that I can't share with certain groups of people that aren't open-minded about that kind of thing. So I feel like I like lose little pieces of my identity when I'm in those groups. And this - this is kind of a place that's very accepting about all aspects of people's identities.”

Participants described the importance of spending time with other youth or adults who understood them and would not judge them. Specifically, TGN youth felt that the time spent together at an overnight camp provided a unique experience that was different compared to other ways they would usually find support, such as online:

“Yeah, so, being specific this year and last year, a lot of people I know, who are like me, I meet like online. So especially non-binary to come here and like, live face-to-face, meet more non-binary people, is always kind of weird because like I've - besides one person where I live - I've never really actually met another non-binary person really, like one my age. So that's nice.”

The in-person connections they built with other youth during the Camp created an environment that made them feel rejuvenated, connected, and supported. As one camper explained, “it’s just kind of refreshing that you could, like, at least for me, you can be around these people, because you know, they’re like you and you know they won’t judge you.”

##### Theme 2: affirming environment created a safe space

TGN campers explained why it was important to spend time in an environment where they were accepted and affirmed in their identities, especially in a space where emphasis was placed on consistent use of correct gender pronouns. Being in a space where “everybody asks your pronouns first” was very important to the campers. Consistent affirmation meant that TGN youth were able to develop confidence in themselves because they had a safe space to be authentic. One camper explained that they felt the experience would have a lasting impact:

“I think being here and hearing other people say that they liked what I had to say, or that they, you know, just being in this environment helped them be themselves. It’s like, walking out of here, I think I’m going to be more willing to share that with people.”

Youth specifically explained how having a safe and affirming space allowed for several specific areas of growth for them. These opportunities for growth were categorized into two subthemes “*An Opportunity to Heal”* and “*Learning Communication Skills,”* which are described in more detail below.

TGN youth described the value, and challenge, of being in a space with others who have experienced similar pain of marginalization. This experience was summarized in the subtheme *An Opportunity to Heal*. The environment that Pride Camp created was a safe space for healing as campers processed painful experiences. One person said, “The past sucks. But it’s like, being here is so much easier to do that.” The camper here is referring to healing through discussing difficult experiences from the past. In summary, youth found it both challenging and valuable to talk face-to-face with others who had similar experiences with bullying or rejection because of their gender identity or expression. As one person explains:

“You have to be willing to dig into that stuff that’s maybe painful and just have an openness to that and that can take a lot of energy, I think, and just like putting yourself out there for people to see and then sort of working through some of those things that we don’t like to talk about.”

Youth appreciated the opportunity that camp created for healing from the many experiences of daily life where they experienced stigma and discrimination for being transgender or gender non-conforming: “Being here at camp reassures you that you’re better than the negative things that have happened to you in the past.”

#### Learning communication skills

TGN youth learned new communication skills that helped them to express themselves and build confidence. Participants described the engagement and improvement in communicating with others, particularly for those who did not know how to talk about their gender identities. For example, one youth said, “We talk a lot about communicating, so the camp is making you more socially comfortable with yourself and talking to others and getting to know people.”

The communication exercises were helpful because they allowed campers to think critically about how and when to share certain aspects of their identities with others. One camper said:

“That's definitely really helpful, and also just being able to learn how to talk to people. Like sharing your story, everybody can tell a story. But we've learned here there's multiple ways you can tell one story. It's all about what you want people to get from your story. So you tell it in different ways.”

After the intervention, campers felt more confident and validated in their gender identities, and in turn, found more comfortable ways to express their identities to others.

With the foundation of a consistently affirming environment, TGN youth developed communication skills that they could take home with them: “They talk a lot about communicating, like actually really try to make you talk to other people, so it’s like making you more socially comfortable with yourself and talking to others and getting to know people.” Another person explained:

“It made me a lot more comfortable and it’s kind of given me ways...it made me have kind of a base for if I have to go to a new class with nobody I know. I have a way to talk to these people and not just be awkward like I usually am.”

Youth explained that feeling accepted and affirmed created a nurturing environment, which helped them share their stories. One camper said:

“Yeah, I think it's really nice, like everyone here is super accepting of everything, and everybody here wants to hear your story, where it's not always the case, or like not everybody wants to hear what you have to say, but here it's not like that. Everybody wants to hear what you have to say and that's really nice.”

Another person said, “The leaders here make it much easier, and not necessarily forcing you, but really encourage you to like speak up, and ensure that you are safe here.”

## Discussion

Pride Camp is one of few immersive, multi-day camp-style programs developed to build resilience and community engagement in LGBTQ youth. Our paper is one of the first to present data on the specific effects of this type of intervention on TGN youth. The findings from quantitative and qualitative data of this pilot study begin to demonstrate how a strength and resilience-based approach to health interventions could be implemented and benefit LGB and/or TGN youth. In the quantitative results, the evidence showed positive changes in self-esteem, resilience, and quality of life among LGBTQ youth between pre- and post-surveys, and similar results for the TGN youth.

Meanwhile, we did not find significant changes in social support or perceived stigma between pre- and post-surveys. Social support was measured by the MSPSS, which is comprised of three domains (family, significant other, and friend support) [16, 32]. Our campers received a moderate level of support from family, within the general population’s normal range of 3–5. This score is significantly higher than its score in a convenience sample of 154 self-identified TGN youth between ages 13 and 21 in Weinhardt et al. [17] This difference could be explained by the fact that written consent was required from legal guardians to participate in the camp. Therefore, family support might be higher among camp participants than among those who were not able to participate.

It is important to note that we did not necessarily expect family support or perceived stigma to change *during* the one-week camp. Perhaps positive family changes occurred once the campers returned home, but we would need a longer follow up period and additional data collection to assess that change. Future studies could involve a family support component in the camp/strength-based intervention to examine its effect.

Additionally, evidence captured in the focus groups further illustrated the ways that Pride Camp provided an affirming and safe environment for transgender and non-binary youth. Consistent with the GMSR model, community connectedness was very important, as described in the first major theme: “Group Energy is Something Special.” Being around other transgender youth was an important component for building this connectedness. Additionally, gender-affirming policies and practices, such as introducing and using pronouns consistently and correctly, ensured that transgender youth were less likely to face stigma or discrimination while they were at camp. Youth explained that this allowed them to further their communication skills and process some of the traumatizing experiences they faced in their lives. These findings are consistent with other research that demonstrates the importance of consistently gender-affirming environments for positive self-image, resilience, and psychological well-being among transgender youth [6, 33]. These qualitative themes were also consistent with the quantitative evidence indicating improvements in self-esteem, resilience, and quality of life. Together, the quantitative and qualitative findings align with minority stress theory that informed the study, suggesting that community connectedness can facilitate resilience and improved well-being.

Another strategy of Pride Camp, based on minority stress theory, was to increase community building and involvement. This was accomplished by providing specific professional development and educational resources for campers (tailored camper programming) and addressing their internal growth, resilience, and mental health. Given that youth might undergo different stages of sexual and/or gender identity development during adolescence, connecting with LGBTQ peers or communities helps them to learn about themselves and develop their identities in a larger social context. For instance, most campers said in the focus groups that being acknowledged as their current/preferred gender identity, by using correct gender pronouns, felt good and supportive.

### Ancillary Benefits

There are several other potential benefits and advantages of this model of Pride Camp. The camp required collaboration between different campus entities and organizations; therefore, campus faculty, staff, and students were primarily the coordinators, and through this experience, became more educated about LGBTQ issues. Also, peer counselors, who were university students, played a key role in facilitating youth’s development of confidence and resilience. Peer counselors were self-identified LGBTQ individuals who had experience developing communication skills and resilience, as well as accepting their own identities. The guidelines for their assistance increased the possibility of creating an LGBTQ-friendly camp environment, which included transgender and non-binary inclusive policies, building role-model relationships, and supporting youth in their transitions from high school to college. Previous studies indicated that poor health among LGBTQ youth was linked to adverse school environments. The 2015 Youth Risk Behavior Survey (YRBS) [34], reported that among self-identified sexual minority students, 34% of them were bullied on school property and 28% were bullied electronically. In a sample of 120 TGN youth in Weinhardt et al. [35], about 46% reported that they experienced discrimination in bathrooms and 56% felt unsafe using bathrooms in public due to their appearance or gender identity. Additionally, studies have found that LGBTQ youth were three times more likely to drop out of high school compared to the general population [33, 36, 37]. Thus, helping LGBTQ youth see a positive future for themselves in a college environment could potentially reduce future socioeconomic consequences, such as underemployment, unemployment, and the likelihood of being involved in the criminal justice system [38, 39].

### Limitations

In addition to having a small sample size for this pilot study, our findings are based on a community sample of LGBTQ youth in a Midwestern urban area. Both the absence of a random sample and the geographic specificity of the intervention should be taken into account when interpreting results. Second, our study is an uncontrolled pre-post design, which limits our ability to make strong causal inferences. Another caveat is that the focus groups were comprised exclusively of transmasculine teens whose sex assigned at birth was female. This does not allow us to include the experiences of transfeminine teens, whose sex assigned at birth was male. The majority transmasculine sample can also be seen as a strength of the study, given that this is an understudied population. Future research should develop rigorous sampling strategies to recruit campers and replicate these findings in various geographical areas. Further research is warranted to understand the experiences and longer-term impacts of Pride Camp on the full range of LGBTQ youth by incorporating a larger sample and controlled design with a longer follow up period to rule out alternative explanations for significant findings, document the longevity of positive effects, and test for potential differences in intervention effects across gender identities and racial/ethnic subgroups.

Additionally, the Camp required parental consent for LGBTQ youth to attend and participate in research. Therefore, youth who were able to attend the camp were required to have some support from their parents for their LGBTQ identity. The potential sampling bias this introduced means we have limited knowledge of family support in those who did not participate and of the potential positive impact of the camp on youth whose parents were less supportive. This impact could be greater than we observed in our sample. Despite these limitations, this study contributes unique data and findings to the literature on this important public health issue, particularly with regard to the potential impact of this type of intervention on transmasculine TGN youth.

## Conclusions

Overall, our pilot findings suggest that LGBTQ youth, including TGN youth, may benefit from the Pride Camp intervention through increased self-esteem, quality of life, and resilience. Some components of the camp, such as providing a gender-inclusive space and community engagement/building, appear to be important in supporting campers’ resilience, sense of solidarity, and self-esteem. These effects are most apparent for youth who are otherwise isolated and oppressed, and who have limited access to LGBTQ resources. Engaging in these experiences appeared to offer them an opportunity for self-discovery, learning, and positive growth under the caring eye of supportive staff and communities. Pride Camp is one of few interventions that is situated to support LGBTQ students at a critical juncture of their educational experience: the transition from high school to college. As a short-term effect, Pride Camp can harness the power and solidarity LGBTQ youth do have to provide respite from the consequences of marginalization. As a long-term effect, the Pride Camp model may foster resilience and psychological well-being among LGBTQ youth in their identity development. The Pride Camp intervention should be tested in a controlled study design and, if effective, encouraged and made available to LGBTQ youth through college campuses widely. It may also be possible to develop a version of the camp that can be delivered online that would convey some of the benefits of the full in-person experience, particularly for participants who cannot attend camp due to geographical distance from a camp setting, social distancing limitations during the COVID-19 pandemic, or other barriers to in-person participation.

## Data Availability

Data are maintained by Lance Weinhardt.
